# Clinical and radiographic results of locking plate with medial support screw in Proximal Humerus fracture – the more, the better?

**DOI:** 10.1186/s12891-024-07700-x

**Published:** 2024-07-24

**Authors:** Jun-Hyuk Lim, Jaeyeon Hwang, Sungmin Kim, Myung-Sun Kim

**Affiliations:** Department of Orthopedic Surgery, Chonnam National University Medical School and Hospital, Gwangju, Republic of Korea

**Keywords:** Proximal humerus fractures, Locking plate, Medial support screw, Open reduction and internal fixation

## Abstract

**Background:**

The calcar of the proximal humerus is a fundamental structure for medial humeral column support. This study aimed to assess the outcome of osteosynthesis across cases of unstable proximal humerus fractures (PHFs) with medial calcar comminution, following treatment with a PHILOS locking plate and medial support screw (MSS).

**Methods:**

Between January 2010 and December 2018, we retrospectively analyzed the outcomes of 121 cases of osteosynthesis for PHFs with medial column disruption. For the medial support, at least one oblique screw was inserted within 5 mm of the subchondral bone in the inferomedial quadrant of the humeral head. All patients were categorized into two groups: 26 patients in the single MSS group, and 95 in the multiple MSS group. Follow-up after at least an year involved clinical and radiographic outcome evaluations, and correspondingly measuring the Constant-Murley score, University of California, Los Angeles (UCLA) shoulder scale, pain visual analogue scale (VAS), major complications, neck-shaft angle (NSA), humeral head height (HHH), and the eventual time to bone union. Risk factors for the major complications were assessed by multivariate logistic regression analyses.

**Results:**

The cohort’s mean age was 64.4 ± 15.4 years, and the mean follow-up duration was 19.5 ± 7.6 months. At the final follow-up, between the single MSS and multiple MSS groups, no significant differences in the Constant-Murley score (*p* = 0.367), UCLA score (*p* = 0.558), VAS (*p* = 0.571), time to bone union (*p* = 0.621), NSA loss (*p* = 0.424), and HHH loss (*p* = 0.364) were observed. The incidence of complications (*p* = 0.446) based on the number of MSS were not significantly different. The initial insufficient reduction after surgery (of NSA < 125°) was found to be a significant risk factor for post-surgical complications.

**Conclusions:**

To treat unstable PHFs, the use of at least one MSS along with a locking plate system is sufficient to achieve satisfactory outcomes. Successful operative treatment using a locking plate for PHF treatment is inherent in anatomical fracture reduction, coupled with medial column support.

## Background

Proximal Humerus Fractures (PHFs) are the most common fractures in the elderly, accounting for 5–6% of all fractures, most prevalently manifesting in patients aged over 65 years [1, 2].

The locking plate in PHF provides stable fixation, prevents fixation failure, and offers biomechanical advantages particularly against osteoporotic fractures [3–6]. Restoring the neck–shaft angle is crucial for preventing fixation failure during plate fixation [7]. To restore the anatomical neck shaft angle, the insertion of a medial supporting screw (MSS) is key. Gardner et al. first demonstrated that, to reduce the fracture and stabilize the medial column support, the inferomedial region of the proximal humerus can be reinforced with screws and the necessary mechanical support [8].

The insertion of an MSS has proven to effectively reduce hospital visits and postoperative surgical complications, especially for complex fractures [9–12]. Surgeons therefore often strive to maximize MSS insertions but challenges may arise due to inappropriate positioning of the plate or when the patient’s humeral head size is small, in cases of inadequate reduction of the humeral head during surgery. Following the methodology proposed by Gardner et al. [8], an MSS is one that is inserted at a specific distance from the subchondral bone, within the inferomedial quadrant of the humeral head. Therefore, even when all the holes of the PHILOS plate (DePuy Synthes, Zuchwil, Switzerland), one of the most widely applied plates for PHFs, are filled with screws, it may be challenging for all screws to be considered as MSSs.

The objective of this study was to evaluate whether a larger number of MSSs indeed affects clinical and radiographic results, reduces major complications, and finally, to realize potential risk factors.

## Methods

The Institutional Review Board of the Chonnam National University Hospital (IRB No. CNUH-2023-351) approved this retrospective study and waived the need for informed consent from the anonymized patients. We identified all consecutive patients with PHFs who underwent open reduction and internal fixation (ORIF) with a PHILOS plate between January 1, 2012, and December 31, 2018, at our department. All procedures were performed by a single shoulder surgeon (MSK) with > 10 years of experience.

We initially identified 195 patients who underwent ORIF using the PHILOS plate for PHFs. We included cases of PHFs with a medial comminution aged twenty years or older, the confirmed presence of at least one MSS, and available follow-up data of a minimum period of 12 months. Patients with no evidence of medial comminution on the initial preoperative radiograph, neurovascular injury at the time of hospital visit, open fractures, or pathological fractures were excluded. Ultimately, 121 patients were included in this study.

### Surgical technique and rehabilitation

**Fig. 1:**
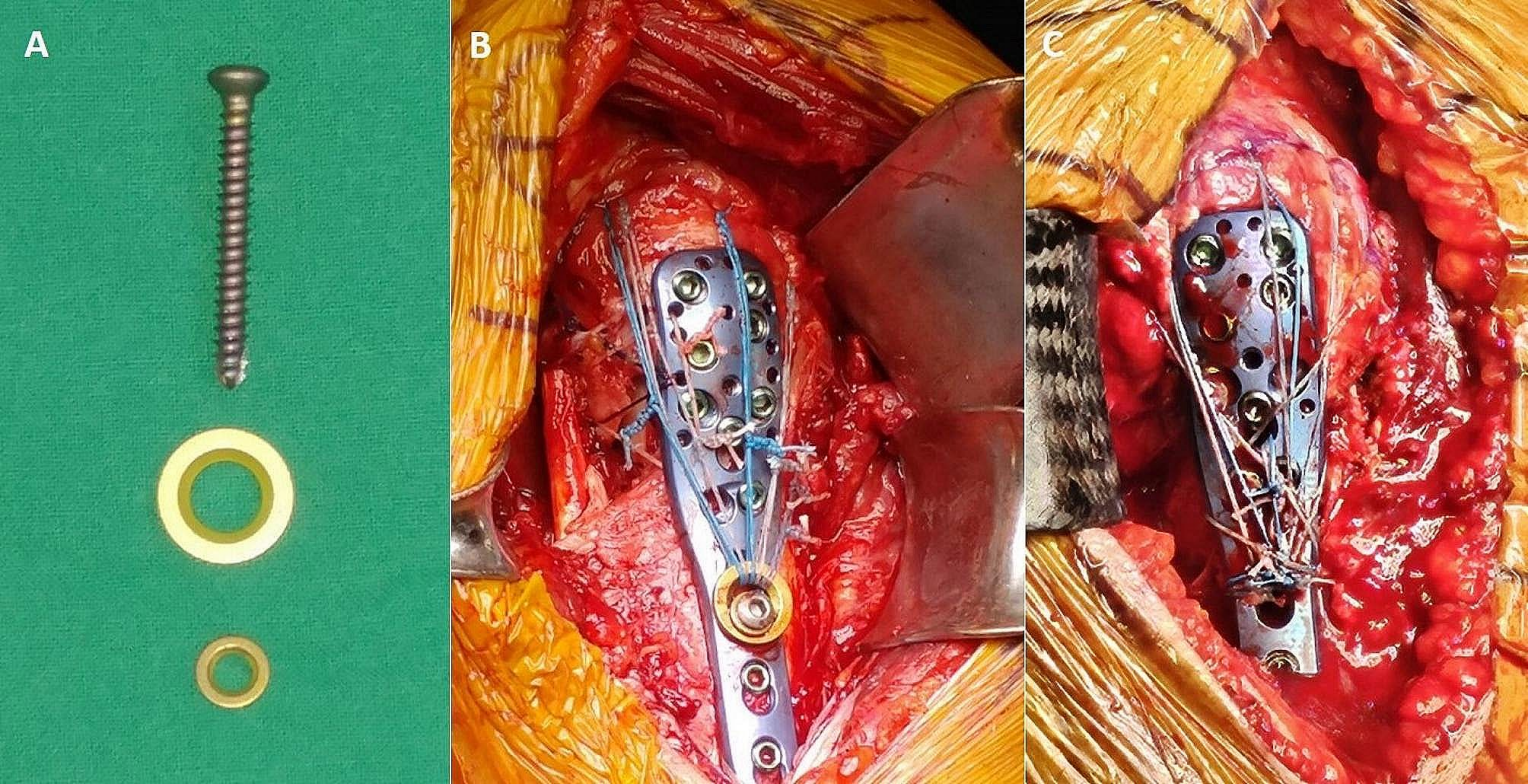
Augmented tension band rotator cuff sutures. **(A)** The conventional screw and two washers through which the FiberWire suture passes. **(B)** The completed appearance of tension band augmentation using conventional screw and two washers. **(C)** The completed appearance of tension band augmentation using eyelet holes and empty screw holes of PHILOS plate

A standard deltopectoral approach was used for all patients under beach chair position. The affected arm was placed on an arm table, allowing easy manipulation and positioning during surgery. Before making a surgical incision, the coracoid process was palpated and marked as a landmark. Then, a surgical incision approximately 10–15 cm in length was made just above the coracoid process, along the anterior aspect, downward along the beginning of the deltopectoral groove, and just above the coracoid process. The pectoralis major and deltoid muscles were located after identifying the deltopectoral groove and cephalic vein, respectively. Subsequently, the deltoid muscle was retracted laterally, and the pectoralis major muscle was retracted medially. Subdeltoid release was performed by finger dissection to create sufficient space for plate placement on the lateral side of the proximal humerus. Once adequate exposure was achieved, the fractured fragments of the proximal humerus were temporarily reduced to their anatomical position using several 1.6-mm Kirschner wires. After confirming well reduction of the fracture fragment using the C-arm, the PHILOS plate was placed on the lateral aspect of the proximal humerus, slightly below the upper end of the greater tuberosity. A 3.5 mm conventional screw was first used with two washers (3.5 mm and 4.5 mm) for contact between the humeral shaft and plate. Subsequently, the MSS were strategically placed within the inferomedial quadrant of the humeral head, and as many locking screws as possible were used to achieve strong fixation. Subsequently, to prevent varus deformity and achieve augmented stability of the humeral head, nonabsorbable sutures (#2 FiberWire suture, Arthrex, Naples, FL, USA) were used to tag the subscapularis, supraspinatus, and infraspinatus tendons. Meanwhile, after removing the 3.5 mm conventional screw, we reinserted the conventional screw 2 mm longer using two washers. Before tightening the conventional screw, we passed the rotator cuff tagging suture through the 4.5 mm washer and pulled it taut, ensuring maximum tension (Fig. 1A and B). After the conventional screw was tightened, a C-arm was used to perform a final assessment to ensure proper reduction and appropriate screw length. However, during the surgery, if the patient’s bone quality is poor, we determined that using locking screws would provide a more stable fixation than conventional screws. Therefore, during the rotator cuff tension band augmentation, nonabsorbable sutures were passed through the eyelet holes or empty screw holes of the PHILOS plate for fixation (Fig. 1C). The surgical field was thoroughly irrigated and a drain was inserted. The deltoid and pectoralis major muscles were reapproximated to their original positions, and the surgical incision was then closed using staples to complete the procedure.

After surgery, the patient wore an L-sling for approximately 4–6 weeks (for immobilization) depending on the preoperative fracture severity. During this period, motion exercises were initiated for the elbows, wrists, and hands. After clinical and radiographic evaluations confirmed complete fracture healing, resistive strengthening exercises were initiated.

### Clinical and radiographic evaluation

We retrospectively conducted clinical and radiographic evaluations of the patients included in the study at 1, 2, 3, 6, and 12 months, postoperatively, and thereafter, at 1-year intervals. Before surgery, we assessed the age, sex, and bone mineral density (BMD) of all the patients. The patient’s BMD was measured according to the WHO bone quality grading [13] by using dual-energy X-ray absorptiometry (DXA) of the hip and lumbar spine to evaluate the T-score. Patients who met the osteoporosis criteria (T-score ≤ − 2.5 standard deviations [SDs]) were categorized into the osteoporosis group. Additionally, to determine the severity of PHF and plan its surgical reduction, preoperative shoulder anteroposterior (AP), scapular Y view, trans-axillary view, and 3-dimensional (3-D) CT scans were obtained for all patients.

Neer’s classification, the prevalent system to assess PHFs severity, is based on four main fracture segments: the head, greater tuberosity, lesser tuberosity, and shaft. Displacement was defined as when each fragment showed more than 1 cm of translation or angulation exceeding 45 degrees [14]. For our study, two authors (SK, JH) independently evaluated fracture severity using shoulder AP radiographs at the time of hospital visit, and categorized the patients into three groups: two-part fracture, three-part fracture, and four-part fracture.

**Fig. 2:**
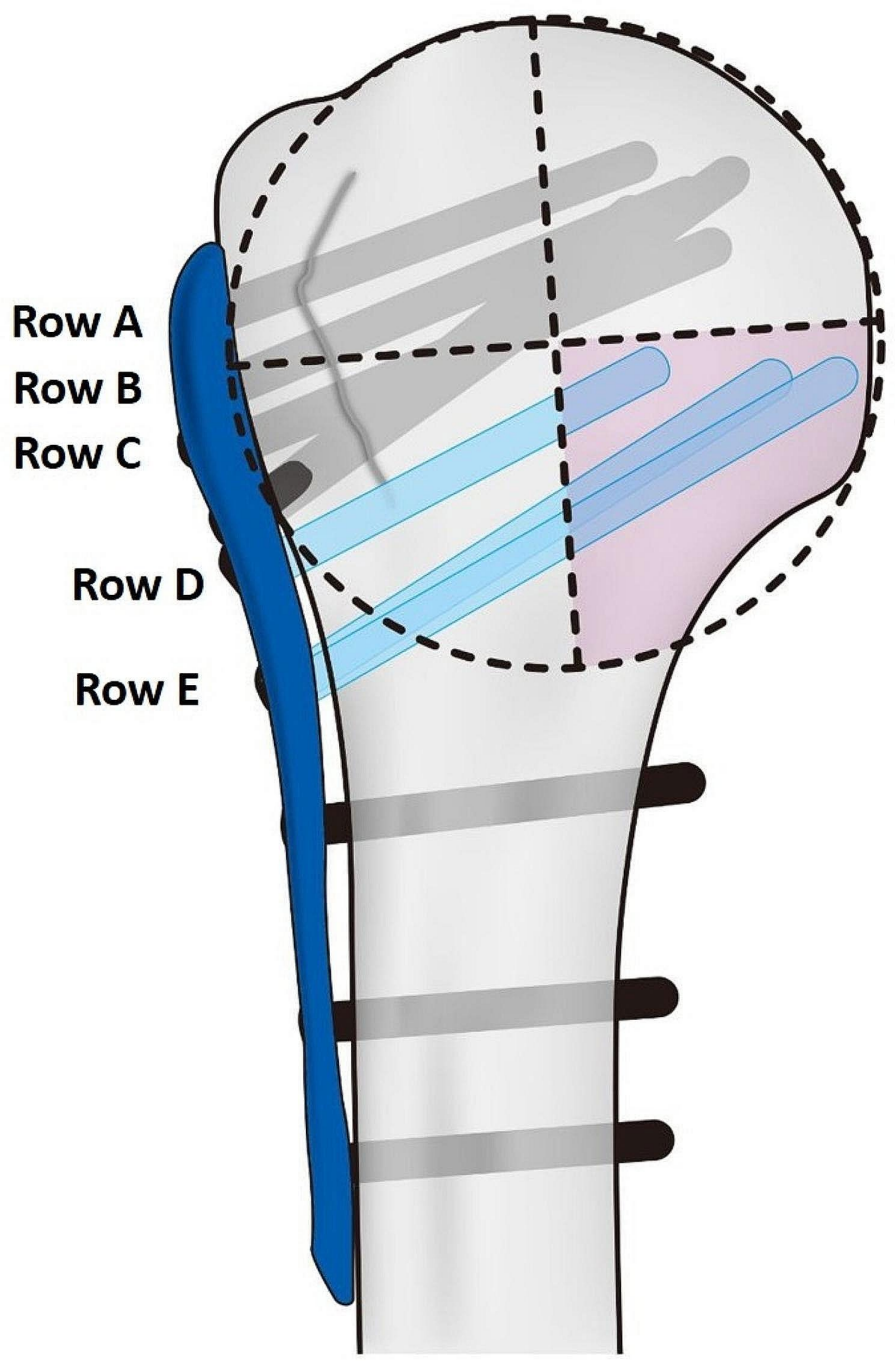
Proximal screw distribution of the PHILOS plate. When positioning the plate appropriately, screws inserted in Rows D and E, among the total of five rows, may be directed towards the inferomedial quadrant of the humeral head. In the true lateral view of the plate, three screws inserted in Rows D and E are oriented towards the inferomedial quadrant of the humeral head

As previously mentioned, we defined the MSS as a screw inserted within the inferomedial quadrant of the humeral head, positioned within 5 mm from the subchondral bone, following the method proposed by Gardner et al. [8]. The proximal screw distribution of the PHILOS plate consists a total of five rows of locking screw holes, labeled A to E. Among these, one hole in row D and two holes in row E are oriented towards the inferomedial quadrant of the humeral head (Fig. 2). Therefore, if the screw is appropriately inserted into any of these three holes, it can be considered as an MSS [15]. The patients were categorized into two groups based on the number of MSS inserted: the Single MSS group, which included patients with one MSS, and the Multiple MSS group, which consisted of patients with two or three MSSs. The classification was determined using the postoperative AP view of the shoulder, as reference.

**Fig. 3:**
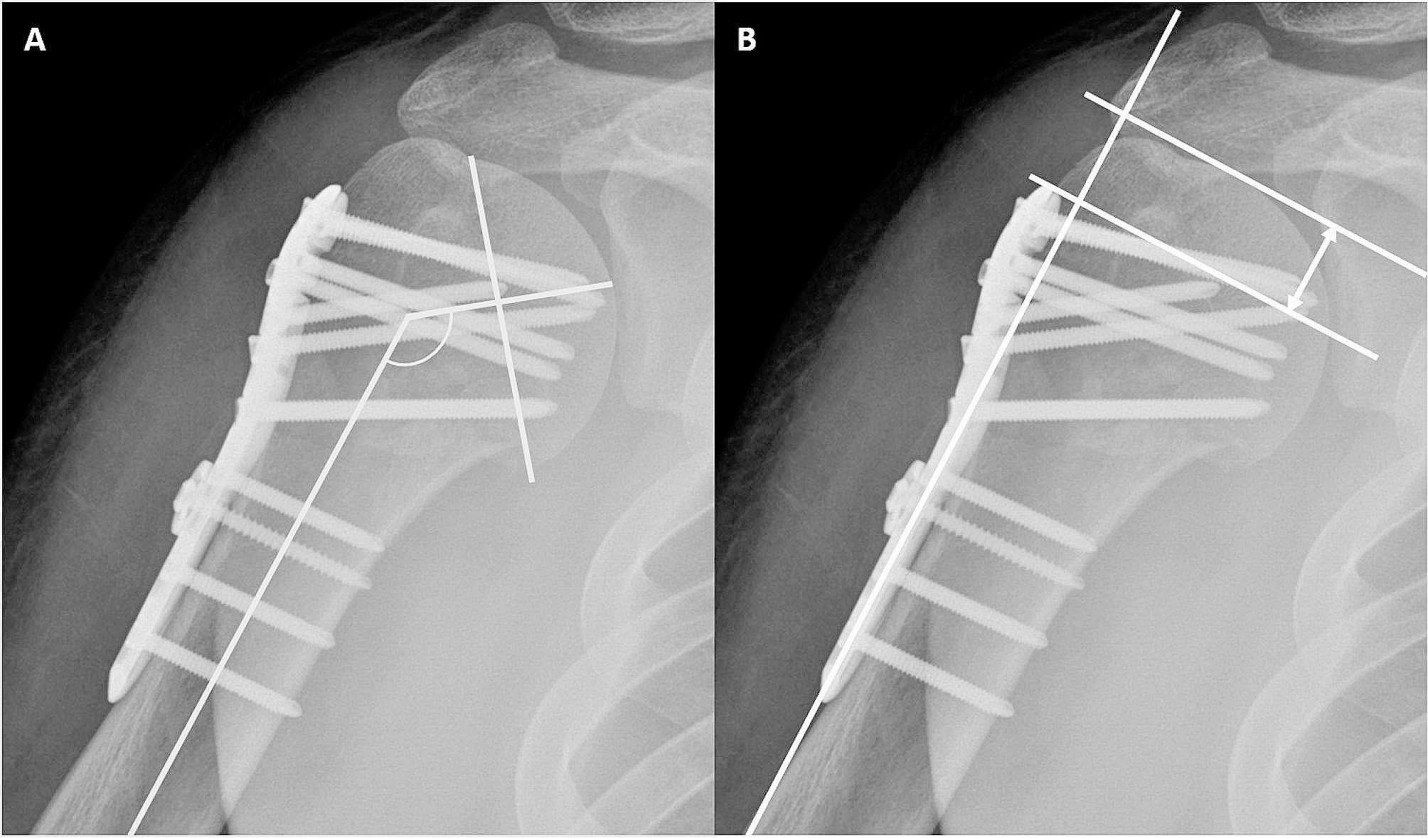
Measurement of Neck-Shaft Angle (NSA) and Humeral Head Height (HHH). **(A)** Measurement of the NSA. The NSA was defined as the angle formed between the central axis of the humeral shaft and a line perpendicular to the anatomical neck of the proximal humerus on a shoulder anteroposterior (AP) view. **(B)** Measurement of the HHH. The HHH was defined by measuring the distance from the top of the plate to the uppermost point of the humeral head, both measured along lines perpendicular to the plate’s axis. The HHH is indicated by a double-headed arrow

To assess radiographic outcomes, the humeral NSA and HHH were measured at each follow-up visit. Postoperatively, NSA and HHH, along with their changes, have been widely regarded as important factors for evaluating functional outcomes and the varus malunion in patients with postoperative PHFs [8, 16–18]. They have been commonly utilized as predictive factors for assessing reduction loss in numerous studies. We defined bony union as the presence of callus formation around the fracture site, which was visible on all X-ray or CT scans during the follow-up period. The NSA was measured on the shoulder AP view using the Paavolainen method, and is defined as the angle between the central axis of the humeral shaft and a line perpendicular to the anatomical neck of the proximal humerus (Fig. 3A) [19]. For the HHH evaluation, we measured the distance between the top of the humeral head and the top of the plate on the shoulder AP view, taking the distance between the two lines perpendicular to the plate axis into account (Fig. 3B) [8]. We calculated the changes in NSA and HHH between the final follow-up and immediate postoperative images. The measurements and confirmation of bony union were performed by two orthopedic surgeons (SK, JH) who were not involved in the clinical care of the patients.

During the follow-up period, we evaluated clinical outcomes related to shoulder function using the Constant-Murley score [20], The University of California-Los Angeles (UCLA) shoulder scale [21], and pain Visual Analog Scale (VAS). Finally, major complications related to PHF were categorized when one or more of the following criteria were met: reduction loss with screw perforation; reduction loss without screw perforation; implant failure; avascular necrosis (AVN); and nonunion.

Based on previous studies, we defined reduction loss as a decrease of > 5 mm in the HHH and > 10° in the NSA [16–18, 22]. Screw perforation was defined as a secondary perforation resulting from incomplete anatomical reduction, excluding screw cutout due to technical errors during surgery [23, 24]. Implant failure was defined as mechanical issues, such as plate or screw breakage, and nonunion was defined as the absence of bony union within six months. Finally, AVN was defined as the presence of evidence of subchondral bone collapse on plain radiographs during the follow-up period, leading to the identification of an irregular joint surface on consecutive follow-up radiographs [25].

### Statistical analysis

We performed Wilcoxon rank-sum tests to compare the means between the single and multiple MSS groups and Fisher’s exact test to compare the major complication rates between the two groups. Additionally, we conducted multivariate logistic regression analysis to identify the risk factors for major complications. Statistical analyses were performed using SPSS^®^ version 25.0 (SPSS, Chicago, IL, USA), with significance defined as *p* < 0.05.

## Results

The patients’ demographic data are summarized in Table 1. The mean age of the patients was 64.4 ± 15.4 years, and the mean follow-up duration was 19.5 ± 7.6 months. Among the 121 patients, 45 (37.2%) were male and 76 (62.8%) were female. Osteoporosis was confirmed in 56 (46.2%) patients. According to Neer’s classification, the fracture severity was classified as two-part in 43 patients (35.5%), three-part in 54 patients (44.6%), and four-part in 24 patients (19.8%). Among the 121 patients, 26 (21.5%) were classified as having a single MSS, and 95 (78.5%) as having multiple MSS.

**Table 1:** Overall patient demographic data

Measures	Single MSS	Multiple MSS	*p-*value
**Number of patients (%)**	26 (21.5%)	95 (78.5%)	
**Age at surgery (year)**	68.3 ± 15.4	63.6 ± 18.2	0.304
**Sex**			0.094
Males	6 (23.1%)	39 (41.1%)	
Females	20 (76.9%)	56 (58.9%)	
**Neer’s classification**			0.257
Two parts	6 (23.1%)	37 (38.9%)	
Three parts	15 (57.7%)	39 (41.1%)	
Four parts	5 (19.2%)	19 (20.0%)	
**Osteoporosis (%)**	13 (50.0%)	42 (44.2%)	0.599
**Follow-up duration (month)**	17.5 ± 7.5	18.1 ± 11.6	0.789

MSS Medial Supporting Screw

During the follow-up period, we compared the clinical and radiographic assessments between the single and multiple MSS groups. At the final follow-up, the clinical assessment showed no statistically significant differences between the two groups in the Constant-Murley score (*p* = 0.367), UCLA shoulder score (*p* = 0.558), and pain VAS score (*p* = 0.571) (Table 2). On radiographic assessment, there were no statistically significant differences between the two groups in the time to bony union (*p* = 0.621), NSA changes (*p* = 0.424), or HHH changes (*p* = 0.364) at the final follow-up (Table 3).

**Table 2:** Clinical outcomes between two groups at final follow-up

	Single MSS	Multiple MSS	*p-*value
**Constant-Murley score**	70.8 ± 10.7	73.1 ± 11.6	0.367
**UCLA shoulder score**	28.0 ± 3.9	29.3 ± 3.2	0.558
**Pain VAS**	1.8 ± 1.5	1.6 ± 1.5	0.571

UCLA University of California, Los Angeles, VAS Visual Analog Scale

**Table 3:** Radiographic outcomes between two groups at final follow-up

	Single MSS	Multiple MSS	*p-*value
**Time to bone union (month)**	4.6 ± 2.2	4.4 ± 1.2	0.621
**Immediately postop NSA (°)**	137.2 ± 6.8	136.3 ± 7.1	0.763
**Final follow-up NSA (°)**	130.3 ± 8.8	130.5 ± 9.7	0.841
**Δ NSA (°)**	6.8 ± 3.9	6.2 ± 4.9	0.424
**Immediately postop HHH (mm)**	14.7 ± 2.7	13.7 ± 4.7	0.360
**Final follow up HHH (mm)**	12.1 ± 2.7	11.2 ± 3.7	0.431
**Δ HHH (mm)**	2.6 ± 1.7	2.5 ± 2.4	0.364

NSA Neck Shaft Angle, HHH Humeral Head Hight

The major complication rates between the single and multiple MSS groups are summarized in Table 4. Among the 121 patients, major complications occurred in ten during the follow-up period. While the multiple MSS group (7.4%) showed a lower proportion of major complications than the single MSS group (11.5%), the difference was not statistically significant (*p* = 0.446). We evaluated whether the number of MSS statistically affected the reduction loss among the complications. Regardless of screw perforation, reduction loss was observed in one case in the single MSS group, and in five cases in the multiple MSS group, and this difference was also not statistically significant (*p* = 1.000).

**Table 4:** Demographic data of major complications

	Single MSS	Multiple MSS	Total	*p-*value
**Complications (%)**	3 (11.5)	7 (7.4)	10 (8.3)	0.446
Reduction loss with screw perforation	1	4	5	
Reduction loss without screw perforation	0	1	1	
Avascular necrosis	1	0	1	
Implant failure	1	1	2	
Nonunion	0	1	1	

Finally, we conducted multivariate logistic regression analysis to identify the risk factors for major complications. Age, sex, osteoporosis, and fracture severity based on Neer’s classification did not differ significantly. However, we found a statistically significant result only when the immediate postoperative NSA was less than 125°, indicating insufficient initial reduction. (*p* = 0.016) (Table 5).

**Table 5:** Multivariate regression analysis affecting risk factors for major complications

Variables	OR (95% CI)	*p*-value
**Age (increase of 1 year)**	0.962(0.904–1.024)	0.229
**Sex (female)**		
Female	1(Reference)	0.328
Male	0.453(0.093–2.212)	
**Osteoporosis**	3.041(0.452–20.455)	0.253
Normal weight	1(Reference)	0.579
Overweight	0.57(0.08–4.13)	0.965
Obesity	1.03(0.27–3.92)	
**Fracture classification**		
Two parts	1(Reference)	
Three parts	0.589(0.08–4.335)	0.603
Four parts	1.221(0.138–10.764)	0.857
**Number of MSS (≥ 2)**	0.434(0.083–2.273)	0.323
**Insufficient reduction (NSA < 125°) at immediate post operation**	8.899(1.660–40.19)	0.016

*CI* confidence interval ^a^The values are given as coefficients with the 95% CI in parentheses

Dependent variable: Development of major complications

MSS Medial Supporting Screw

## Discussion

MSS insertion is crucial for providing stable medial column support during locking plate fixation for PHF with concomitant medial comminution. Since Gardner et al. highlighted the significance of the MSS [8], numerous studies have reported that the insertion of the MSS prevents postoperative complications related to fractures and contributes to better functional and radiographic outcomes by maintaining a better reduction [9, 11, 12]. However, to the best of our knowledge, there are few clinical and radiographic studies related to the number of MSS. We aimed therefore to investigate the clinical and radiographic outcomes and major complication rates based on the number of MSS.

**Fig. 4:**
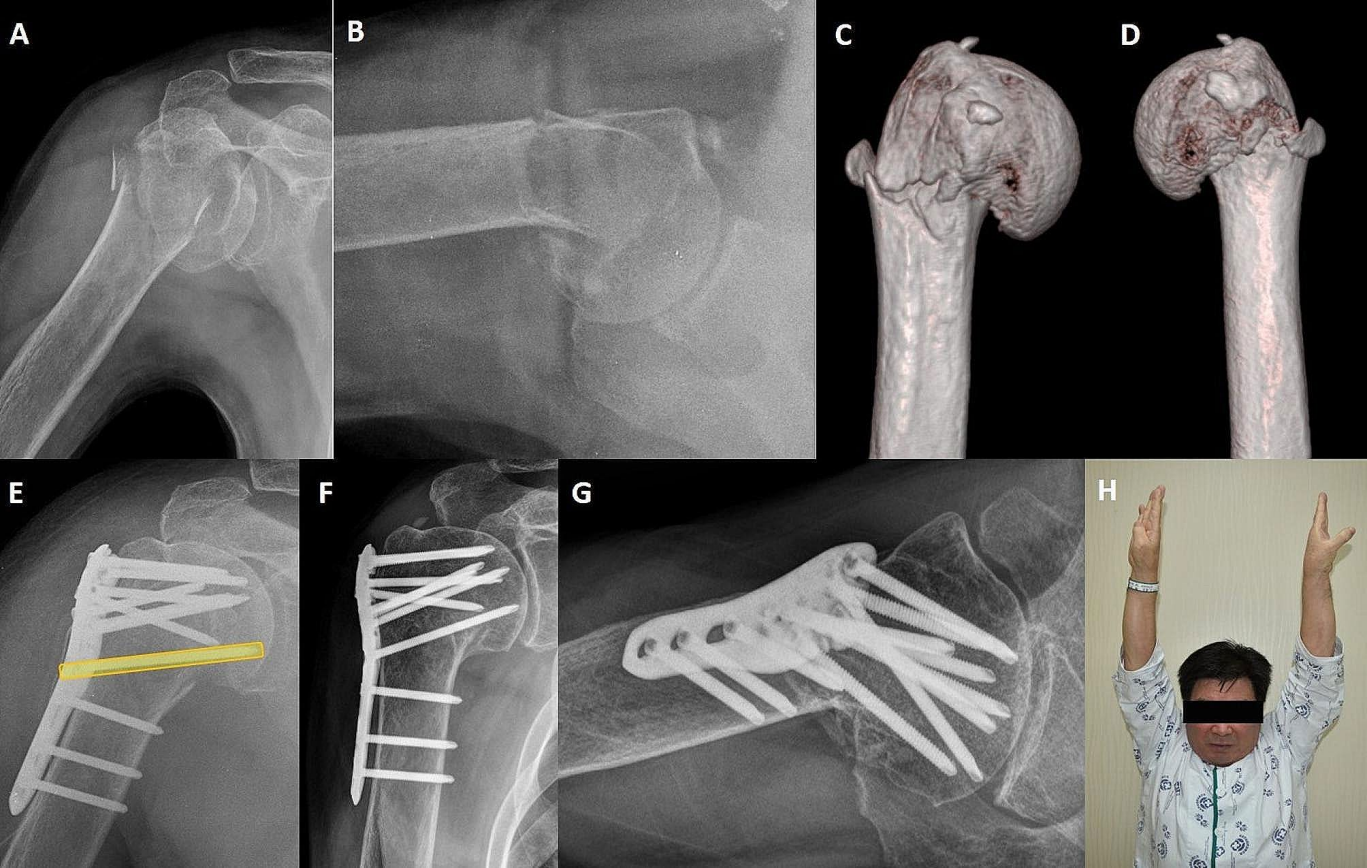
A case demonstrating favorable clinical and radiographic outcomes with a single medial supporting screw (MSS). A 71-year-old male patient sustained a right shoulder injury from a fall. The shoulder plain radiograph shows a right proximal humerus fracture (PHF) classified as a two-part fracture according to Neer’s classification in the **(A)** shoulder AP and **(B)** trans-axillary views. The **(C)** anterior and **(D)** posterior views of the 3D CT images reveal varus angulation of the proximal humerus accompanied by medial comminution. The **(E)** immediate postoperative radiograph shows the insertion of a single MSS. At 1 year postoperatively, **(F)** shoulder AP and **(G)** trans-axillary views show complete bony union at the fracture site without complications. **(H)** The patient exhibits a good range of motion comparable to the unaffected side at 1 year postoperatively

In our study, there was no significant difference between single and multiple MSSs in terms of clinical and radiographic results or major complication rates (Fig. 4A–G). Zeng et al. suggested that the number of MSS could impact mechanical stability and that a greater number of MSS could improve shoulder function [15]. Similar to our finding, despite the statistically non-significant results, the multiple MSS group had a lower rate of major complications. However, our study differs from that of Zeng et al. in that we used tension-band rotator cuff sutures in all patients to achieve additional stability. Several authors have discussed that additional tension-band suture fixation lowers fracture-related complications and improves functional outcomes [26, 27]. Cho et al. introduced a robust tension band suture fixation method utilizing two washers, 3.5 mm and 4.5 mm in size, to ensure a strong tensile force is transmitted through the 4.5 mm washer hole [28]. We used this technique for patients with relatively good bone quality, while for those with poorer bone quality, we performed tension-band suture fixation using the eyelet holes or empty screw holes of the PHILOS plate. Tension band suture augmentation provided additional mechanical stability, and prevented reduction loss even in the single MSS group in our study.

**Fig. 5:**
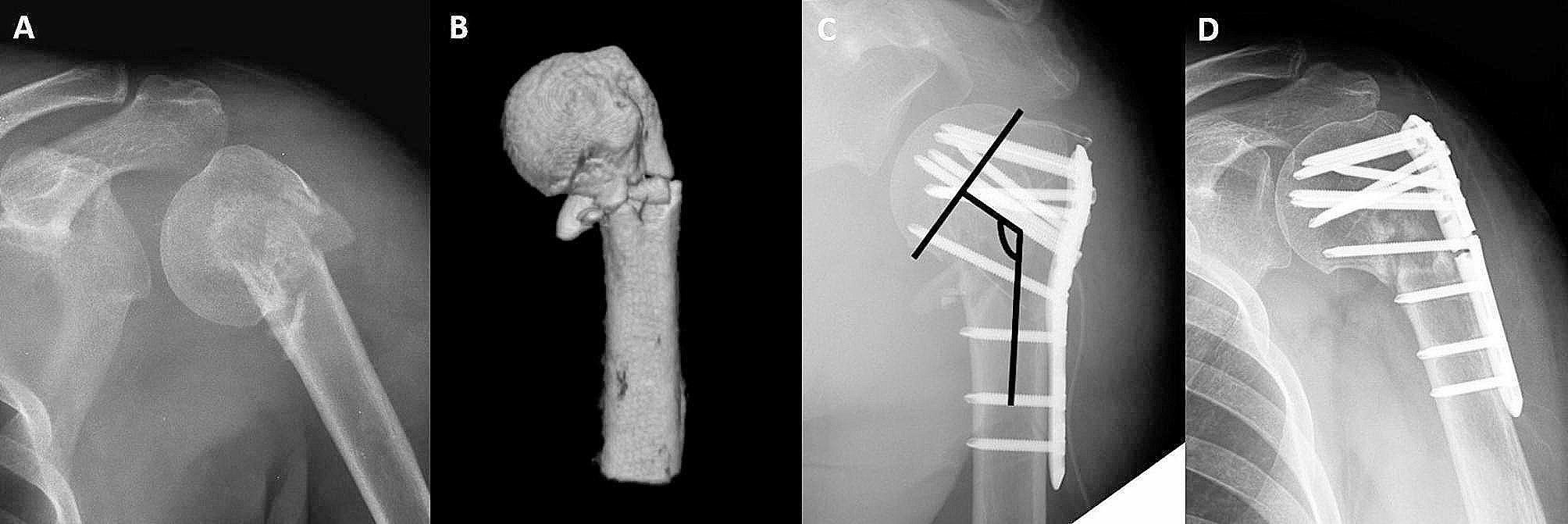
A case demonstrating implant failure, a major complication. A 62-year-old female patient woman sustained a left shoulder injury from slipping down. The shoulder plain radiograph shows a left proximal humerus fracture (PHF), classified as a Neer two-part fracture. **(A)** Shoulder AP and **(B)** shoulder 3D CT show medial comminution. **(C)** Immediate postoperative shoulder AP view shows that one medial supporting screw (MSS) is used, and the immediate postoperative neck–shaft angle (NSA) is 121º. **(D)** At 1 year postoperatively, implant breakage is observed

Multivariate logistic regression analysis revealed a statistically significant increase in the major complication rate when the immediate postoperative NSA was less than 125° (Fig. 5A–D). Several authors have emphasized that the postoperative NSA is a key factor in reduction loss [7, 16, 18, 22]. Wang et al. particularly underscored the importance of maintaining the NSA between 130° to 150° during surgery as the most crucial element for the optimal positioning of the MSS [7]. Our study aligns with previous studies in this regard, emphasizing the significance of meticulous preservation of the NSA during reduction as a critical factor in potentially mitigating future complication rates. Meanwhile, several cadaveric biomechanical studies have suggested that increasing the number of screws directed towards the head fragment can prevent complications such as varus collapse of the humeral head [29–31]. Given these factors, surgeons may attempt to maximize MSS insertion during locking plate fixation of PHFs. However, contrary to the surgeons’ intentions, it is not always feasible to insert as many MSSs as desired. Instead, it may be crucial to strive to maintain the NSA at an appropriate angle during intraoperative procedures and to ensure that the positioning of the plate is neither excessively proximal nor distal to make the maximal insertion of MSSs. Recent studies too have suggested that the plate should not be positioned too proximally, and that positioning screws as inferiorly as possible enhances reduction quality [32, 33].

In our study, age, sex, osteoporosis, and fracture severity were not identified as risk factors for major complications. Taskesen et al. concluded that osteoporosis parameters vary across age and sex among patients with PHF, and that osteoporosis did not emerge as the primary factor that significantly influences fracture type and surgical outcomes [34]. Our study showed similar findings, possibly due to the diverse age range of the patients in our study group, with more than half not falling into the osteoporosis group, based on BMD measurements. These factors may have influenced the results; therefore, further studies are warranted.

Based on the results of our study, we suggest that all surgeons opt for locking plate fixation for PHFs with concomitant medial comminution, and that inserting only one MSS may not necessarily lead to reduction loss and unfavorable shoulder functional outcomes. A recent study reported various methods to ensure additional reduction stability, such as using an intramedullary fibular strut allograft [35–37] or calcium sulfate augmentation [38]. We believe that if more than one MSS can be inserted during locking plate fixation, sufficient stability can be achieved for postoperative rehabilitation and patient education, leading to a successful union of the fracture. However, comprehensive preoperative planning is essential when treating patients with PHF and medial comminution. Preoperative analysis of fracture severity and patterns using 3D-CT can be beneficial. When dealing with severe medial comminution of PHFs, in which obtaining stability with only one MSS may be challenging, the aforementioned approaches could serve as favorable options for achieving positive clinical and radiographic outcomes. Lastly, we emphasize the importance of verifying the NSA with fluoroscopy during surgery. If the NSA is less than 125º, the surgery should not be completed as maximizing the restoration of the NSA is crucial for preventing major complications after surgery. To achieve optimal NSA restoration, surgeons can additionally consider options such as intramedullary fibular strut allografts and calcium sulfate augmentation.

Our study had several limitations. First, this was a single-center, retrospective study. Second, the non-standardized number of screws directed towards the proximal humeral head fragment, excluding the MSS, may have influenced the outcomes. Third, this study employed a specific type of plate (PHILOS plate), and variations in the results could arise from studies that utilized different types of locking plates. Finally, the relatively limited duration of patient follow-up poses difficulties in assessing survival rates and long-term outcomes. Long-term prospective studies are required to confirm these findings. Despite these limitations, our study holds value as being the first to demonstrate that, despite the limitations posed by a single MSS insertion (during locking plate fixation for PHF with concomitant medial comminution), it does not yield clinically or radiographically inferior results compared with multiple MSS insertions until the point of bony union, contrary to the valid concerns of surgeons.

## Conclusion

In conclusion, when treating unstable PHFs with concomitant medial comminution using locking plate fixation, the insertion of at least one MSS can lead to clinically and radiographically satisfactory outcomes. The number of MSS cases did not have a significant impact on clinical and radiographic outcomes, but striving to maintain the NSA at an appropriate angle is key. Moreover, an insufficient immediate postoperative NSA remains the only significant risk factor for major complications.

## Data Availability

The datasets used and/or analyzed during the current study are available from the corresponding author on reasonable request.

## Abbreviations

3-D: 3-dimensional
AP: Anteroposterior
BMD: Bone mineral density
HHH: Humeral head height
MSS: Medial support screw
NSA: Neck-shaft angle
ORIF: Open Reduction and Internal Fixation
PHFs: Proximal humerus fractures
UCLA: University of California, Los Angeles
VAS: Pain visual analogue scale
